# IL‐31 transgenic mice show reduced allergen‐induced lung inflammation

**DOI:** 10.1002/eji.202048547

**Published:** 2020-07-21

**Authors:** Theresa Neuper, Daniel Neureiter, Muamera Sarajlic, Helen Strandt, Renate Bauer, Harald Schwarz, Patrick Suchanek, Evgeniia Korotchenko, Stacey R. Dillon, Peter Hammerl, Angelika Stoecklinger, Richard Weiss, Jutta Horejs‐Hoeck

**Affiliations:** ^1^ Department of Biosciences University of Salzburg Salzburg Austria; ^2^ Institute of Pathology Paracelsus Medical University/Salzburger Landeskliniken (SALK) Salzburg Austria; ^3^ ZymoGenetics, Inc. Bristol‐Myers Squibb East Syracuse NY USA

**Keywords:** Grass pollen allergen, IL‐31, IL‐31RA expression, Leukocyte infiltration, Lung inflammation

## Abstract

Interleukin‐31 (IL‐31) is a Th2 cell–derived cytokine that has been closely linked to pruritic skin inflammation. More recently, enhanced IL‐31 serum levels have also been observed in patients with allergic rhinitis and allergic asthma. Therefore, the main aim of this study was to unravel the contribution of IL‐31 to allergen‐induced lung inflammation. We analyzed lung inflammation in response to the timothy grass (*Phleum pratense*) pollen allergen Phl p 5 in C57BL/6 wild‐type (wt) mice, IL‐31 transgenic (IL‐31tg) mice, and IL‐31 receptor alpha‐deficient animals (IL‐31RA^−/−^). IL‐31 and IL‐31RA levels were monitored by qRT‐PCR. Cellular infiltrate in bronchoalveolar lavage fluid (BALF) and lung tissue inflammation, mucus production as well as epithelial thickness were measured by flow cytometry and histomorphology. While allergen challenge induced IL‐31RA expression in lung tissue of wt and IL‐31tg mice, high IL‐31 expression was exclusively observed in lung tissue of IL‐31tg mice. Upon Phl p 5 challenge, IL‐31tg mice showed reduced numbers of leukocytes and eosinophils in BALF and lung tissue as well as diminished mucin expression and less pronounced epithelial thickening compared to IL‐31RA^−/−^ or wt animals. These findings suggest that the IL‐31/IL‐31RA axis may regulate local, allergen‐induced inflammation in the lungs.

## Graphical abstract

This study describes a role for IL‐31 signaling in the context of allergen‐induced lung inflammation. Mice overexpressing IL‐31 develop less severe lung inflammation upon allergen exposure, characterized by decreased leukocyte infiltration, mucus production and epithelial thickening. Thus, our data indicate that IL‐31 signaling regulates allergen‐induced lung inflammation.

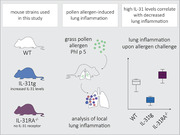

## Introduction

Interleukin‐31 (IL‐31) is an IL‐4‐dependent Th cell–derived cytokine [1, 2, 3], which is well recognized as an important player in pruritic skin diseases [1, 4, 5]. In humans, enhanced IL‐31 expression is positively correlated with the pathogenesis of allergic skin disorders, including atopic dermatitis and allergic contact dermatitis [5, 6, 7]. Accordingly, mice overexpressing IL‐31 show a strong Th2‐associated inflammatory skin phenotype [1]. As it was suggested that IL‐31 may evoke itch by acting on IL‐31RA‐expressing neurons [4], blocking IL‐31/IL‐31RA interactions has been proposed as a strategy to alleviate Th2‐mediated itch in order to allow patients to break out of the vicious circle of itching and scratching. The first human studies using humanized antibodies targeting IL‐31RA, the ligand‐binding subunit of the heterodimeric IL‐31 receptor complex, showed that blocking IL‐31 signaling significantly improves pruritus in patients with atopic eczema [8, 9, 10]. In addition to human keratinocytes, expression of the IL‐31 receptor complex has also been observed on human DCs [11], eosinophils [12], and primary human bronchial and alveolar epithelial cells [13], suggesting that the lungs might also be an important target site of IL‐31. Indeed, compared to healthy controls, patients with asthma or allergic rhinitis showed higher serum IL‐31 concentrations, which correlated positively with Th2‐related cytokines and disease severity [14, 15, 16].

A recent phase 2b randomized study reports that atopic dermatitis patients treated with the anti‐IL‐31RA antibody nemolizumab showed rapid and significant improvement of pruritus and a reduction in skin lesions, with nasopharyngitis and asthma‐like events in patients with preexisting asthma being the most common side effects in this study [10]. This indicates that blocking of IL‐31RA may induce distinct outcomes in skin and lung. However, the contribution of IL‐31 to inflammatory responses in the lungs is not well understood.

## Results and discussion

### Phl p 5 immunization and challenge induce IL‐31RA expression in lung tissue

**Figure 1:**
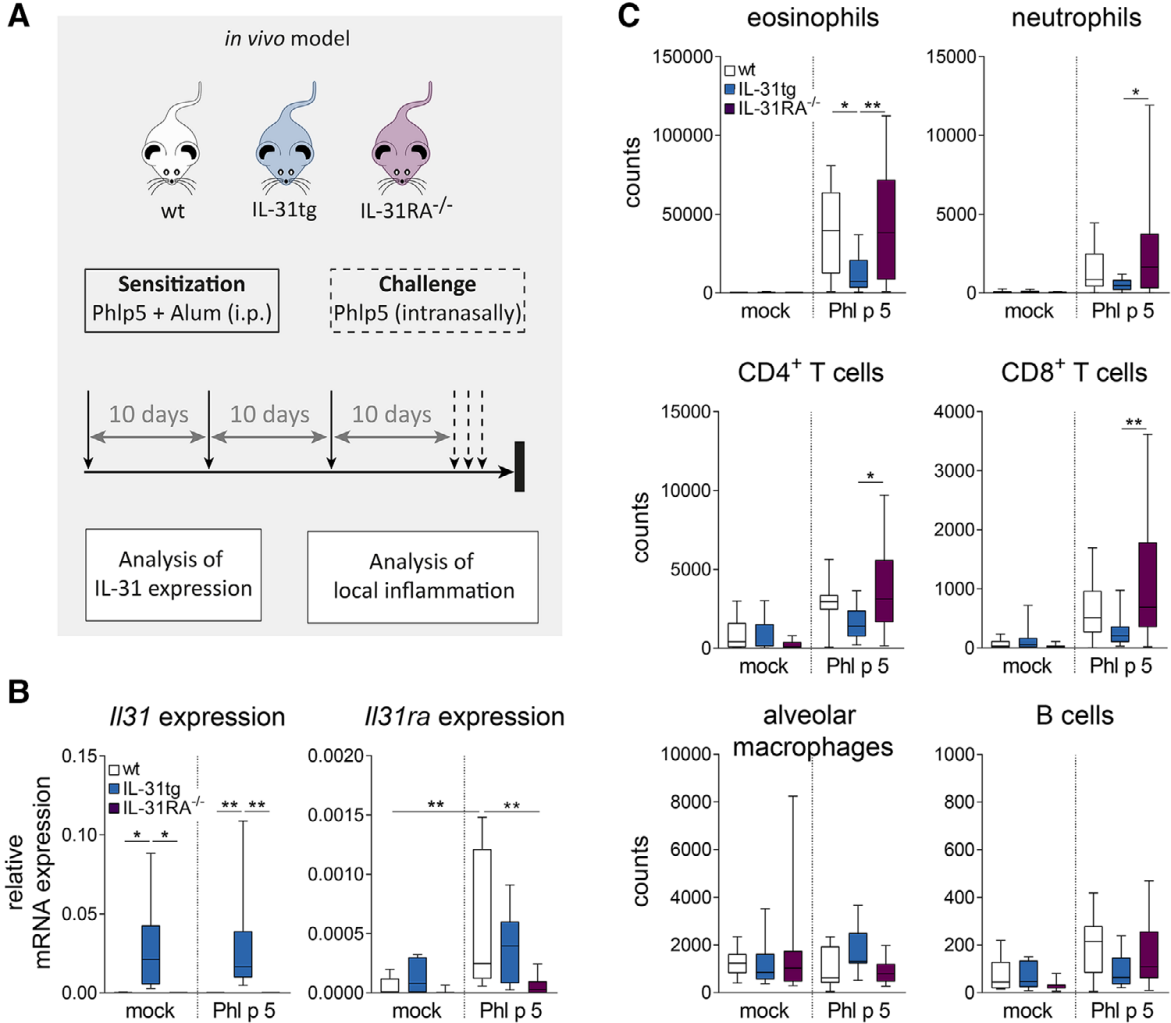
IL‐31RA expression and leukocyte infiltration in a model of Phl p 5‐induced allergic lung inflammation. (A) Schematic overview and time scale describing allergic lung inflammation induced by the grass pollen allergen Phl p 5 in C57/Bl6 wild‐type (wt), IL‐31 transgenic (IL‐31tg), and IL‐31 receptor alpha‐deficient (IL‐31RA^−/−^) mice. For sensitization, mice were injected with 1 μg recombinant Phl p 5 absorbed to alum three times intraperitoneally at 10‐day intervals. Ten days after the last allergen immunization, mice were challenged by intranasal instillation of 1 μg of Phl p 5 dissolved in PBS on three consecutive days. (B) *Il31* and *Il31ra* mRNA expression was analyzed by qPCR in lung tissue of wt, IL‐31tg, and IL‐31RA^−/−^ animals after mock or allergen challenge. Box plots represent median and min to max (two experiments, *n* = 9–10). (C) BALF of mock or allergen‐challenged animals was harvested. Counts of eosinophils, neutrophils, monocytes, CD4^+^ cells, CD8^+^ cells, and B cells were determined by flow cytometry. Box plots represent median and min to max (three experiments, *n* = 11–17). For statistical analysis, one‐way ANOVA with Tukey's posthoc test was performed: *p < 0.05, **p < 0.01, ***p < 0.001.

To elucidate the role of IL‐31 in allergen‐mediated lung inflammation, we investigated allergen‐dependent immune responses induced by the timothy grass (*Phleum pratense*) pollen allergen Phl p 5 in wild type (wt), IL‐31RA‐deficient (IL‐31RA^−/−^), and IL‐31‐constitutively expressing transgenic (IL‐31tg) mice, which express significantly higher levels of IL‐31 in various tissues, including the lungs (Supporting Information Fig. S1). Female IL‐31tg, IL‐31RA^−/−^, and wt mice were immunized three times with recombinant Phl p 5 at 10‐day intervals, followed by an allergen challenge 10 days after the last immunization (Fig. 1A). While IL‐31 expression was clearly enhanced in lungs of mock‐treated IL‐31tg mice compared to wt and IL‐31RA^−/−^ mice, IL‐31RA levels were barely detectable in mock‐treated mice of all three strains (Fig. 1B). Following Phl p 5 challenge, IL‐31 expression was not further induced, but we observed clearly elevated expression of IL‐31RA in lung tissue of wt as well as IL‐31tg mice (Fig. 1B). These data indicate that allergen administration results in increased IL‐31RA expression in the lungs and raise the question of the potential role of IL‐31 signaling during local lung inflammation.

### IL‐31 transgenic mice show less pronounced signs of allergen‐induced lung inflammation

**Figure 2:**
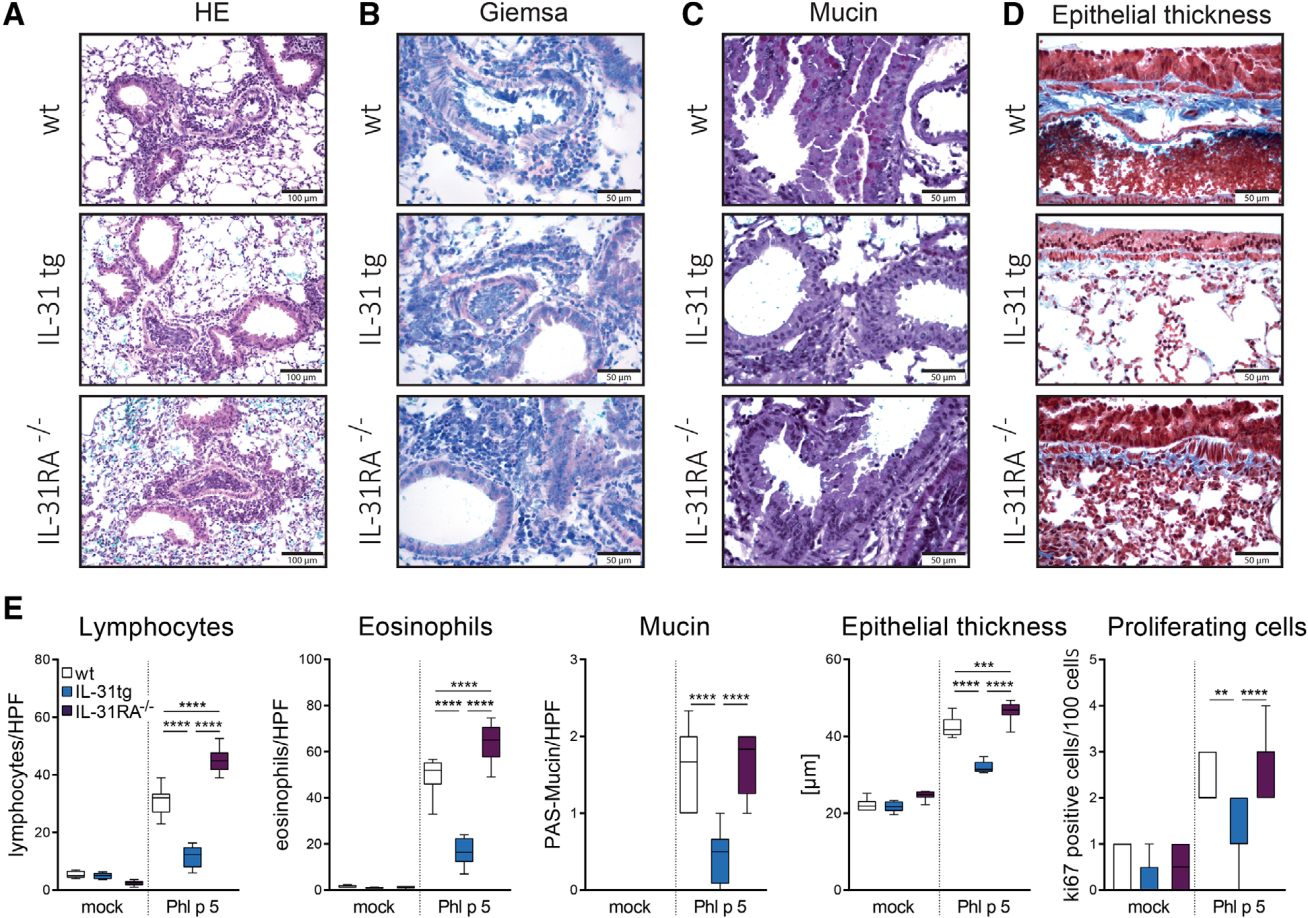
Analysis of histopathology and cellular infiltration in wt, IL‐31tg, and IL‐31RA^−/−^ mice after mock or Phl p 5 challenge. Perfused lungs of Phl p 5‐challenged mice were stained with (A) H&E to assess histomorphology (scale bar, 100 μm), (B) Giemsa to identify infiltrating lymphocytes and eosinophils (scale bar, 50 μm), (C) PAS to determine mucus production (scale bar, 50 μm), or (D) trichrome staining to monitor epithelial thickness (scale bar, 50 μm). (E) Numbers of lung‐infiltrating lymphocytes and eosinophils as well as proliferating epithelial cells and levels of mucin production and epithelial thickness were assessed. Box plots represent median values (+ min to max) of five to ten mice per group (two experiments, *n* = 5–10). For statistical analysis, one‐way ANOVA with Tukey's posthoc test was performed. **p < 0.01, ***p < 0.001, ****p < 0.0001.

The fact that IL‐31RA was upregulated in lung tissue upon Phl p 5 challenge suggests a role of IL‐31 in local lung inflammation. Therefore, we monitored cell composition in bronchoalveolar lavage fluid (BALF) as well as histomorphological changes after intranasal Phl p 5 challenge. Compared to mock‐challenged animals, we observed substantial increases in the numbers of eosinophils, neutrophils, CD4^+^, and CD8^+^ T cells in the BALF of Phl p 5‐challenged mice, with eosinophils representing the majority of leukocytes in each of the three genotypes (wt, IL‐31tg, and IL‐31RA^−/−^) (Fig. 1C). Of note, BALF of IL‐31tg mice showed significantly lower infiltration of eosinophils, neutrophils, CD4^+^, and CD8^+^ T cells compared to BALF of IL‐31RA^−/−^ mice, and eosinophil counts were also significantly reduced compared to wt mice (Fig. 1C). In accordance, H&E (Fig. 2A) and Giemsa staining (Fig. 2B) revealed significantly lower numbers of lymphocytes and eosinophils in the lung tissue of IL‐31tg mice compared to wt and IL‐31RA^−/‐^ mice (Fig. 2E). However, no significant difference in IL‐5 and IL‐13 secretion by Phl p 5 restimulated splenocytes of all three strains was detected, suggesting that IL‐31 does not specifically contribute to systemic responses induced by Phl p 5 (Supporting Information Fig. S2). To substantiate the hypothesis that high IL‐31 levels show a negative correlation with local lung inflammation, we monitored mucus production and epithelial thickness. Upon allergen administration, IL‐31tg mice produced significantly lower amounts of mucus compared to wt and IL‐31RA^−/‐^ mice (Fig. 2C and E). Moreover, thickening of the epithelium was not as pronounced in IL‐31tg mice as observed in wt and IL‐31RA^−/−^ animals (Fig. 2D and E), and this result correlates with lower numbers of proliferating cells within the epithelium of IL‐31tg mice (Fig. 2E). These findings strengthen the hypothesis that high levels of IL‐31 reduce allergen‐induced lung inflammation.

Previous studies have already described a limiting role for IL‐31/IL‐31RA in Th2 inflammation [17, 18]. In the latter studies, inflammation in the lung and in the gut was induced by *Schistosoma mansoni eggs* and the gastrointestinal helminth *Trichuris muris*, respectively. In both models, IL‐31RA deficiency was clearly correlated with exacerbated type‐2 inflammatory responses, suggesting that functional IL‐31–IL‐31R interactions may keep type‐2 inflammation in the lungs and the intestine under control. Similar findings were described in a recent study where ovalbumin (OVA)‐induced allergic asthma was investigated in IL‐31RA^−/−^ mice [19]. This study shows that functional IL‐31 signaling protects against overshooting inflammation, not only in a model of parasite‐induced Th2 inflammation but also in Th2 inflammation induced by the model allergen OVA. This conclusion also holds true in our model, which involves the clinically relevant grass pollen allergen Phl p 5 and confirms the inflammation‐limiting effects of IL‐31 in IL‐31tg mice. Minor differences in the severity of allergic lung inflammation observed between the two allergen‐induced models and the fact that in our study IL‐31 was not significantly induced upon Phl p 5 challenge may be explained by the different protocols for sensitization and allergen challenge. While we used 1 μg of allergen for sensitization and challenged the mice on three consecutive days, Huang and colleagues used 100 μg of OVA for sensitization and challenged the mice on seven consecutive days. Although the detailed mechanisms underlying IL‐31 counter‐regulation of lung inflammation are not yet fully understood, earlier studies demonstrated that IL‐31RA^−/−^ mice are hyperresponsive to oncostatin M (OSM) [20]. Similar to IL‐31, OSM is a member of the gp130 family of cytokines and binds to OSMRB as does IL‐31 [21]. It was suggested that the excess of OSMRB receptor chains in IL‐31RA^−/−^ mice leads to overresponsiveness to OSM, which can result in enhanced lung inflammation mediated by the OSM‐inducible factors IL‐6 and VEGF [22, 23]. Vice versa, high levels of IL‐31, as observed in the IL‐31tg mouse, might compete with OSM for receptor binding and thereby suppress OSM‐mediated inflammatory responses in the lungs.

## Concluding remarks

Consistent with other reports, this study demonstrates that IL‐31/IL‐31RA interactions protect against allergen‐induced local lung inflammation. Compared to wt controls and IL‐31RA^−/‐^ animals, mice overexpressing IL‐31 show diminished leukocyte infiltration into the lungs, reduced mucus production, and a decrease in epithelial thickening, suggesting that functional IL‐31 signaling might ameliorate lung inflammation induced by the grass pollen allergen Phl p 5.

## Materials and methods

### Mice, immunization, and intranasal challenge

Animal experiments were conducted in accordance with EU guidelines 86/609/EWG and national legal regulations (TVG 2012) and all efforts were made to minimize or avoid suffering. Experiments were approved by the Austrian Ministry of Science (permit numbers: BMWFW‐66.012/0025‐WF/V/3b/2015, BMWFW‐66.012/0034‐WF/V/3b/2017). IL‐31RA^−/−^ mice and IL‐31tg mice constitutively expressing IL‐31 under the transcriptional control of the murine Lck promoter [1] were obtained from ZymoGenetics/Bristol‐Myers Squibb (Seattle, USA) and housed under specifc pathogen‐free (SPF) conditions at the local animal laboratories of the University of Salzburg. The IL‐31tg line was maintained by breeding IL‐31tg males to C57BL/6N females. Four‐ to six‐month‐old IL‐31tg offspring and transgene‐negative littermates as well as IL31RA^−/−^ mice were used for the experiments. None of the animals showed signs of skin inflammation at the time of immunization and allergen challenge.

Female wt, IL‐31tg, and IL‐31RA^−/−^ mice were immunized intraperitoneally three times at 10‐day intervals with 1 μg of recombinant Phl p 5 in sterile phosphate‐buffered saline (PBS) with 100 μL of Al(OH)_3_ (alum; Serva, Heidelberg, Germany) in a total volume of 200 μL. Control animals were injected with sterile PBS with 100 μL of Al(OH)_3_ without allergen. Recombinant Phl p 5 was expressed in *Escherichia coli* BL21 (DE3) and purified as described previously [24]. Endotoxin removal was performed by using EndoTrap® red (Hyglos). Endotoxin content was evaluated by Limulus amebocyte lysate assay (Cape Cod) according to the manufacturer's instructions and by a cell‐based detection assay as described by Schwarz et al. [25] (Supporting Information Fig. S3). Proteins used for all experiments had >95% purity (SDS‐PAGE), and the endotoxin content was lower than 0.3 pg/μg. Ten days after the last immunization, lung inflammation was induced by intranasal instillation of 1 μg of Phl p 5 in 40 μL of PBS divided between the two nostrils on three consecutive days. Control mice received 40 μL of PBS without allergen.

### Analysis of bronchoalveolar lavage specimens

Bronchoalveolar lavage was performed after cervical dislocation of the mice by introducing 2 × 1 mL of ice‐cold PBS into the trachea using a catheter and syringe. For flow cytometry analysis, we adhered to the guidelines for the use of flow cytometry [26]. Lavaged cells were stained with CD45 PerCP‐Cy5.5 (30‐F11; Biolegend), Ly‐6G APC (1A8‐Ly6g; Thermo Fisher), CD4 eFluor 450 (GK1.5; Thermo Fisher), CD8a FITC (53‐6.7; Thermo Fisher), Siglec F PE (E50‐2440; BD Biosciences), and CD19 PE‐Cy7 (6D5; Biolegend). Eosinophils were distinguished from other leukocytes by their Gr1^low^ Siglec F^high^ BV510‐autofluorescence^low^ phenotype (see Supporting Information Fig. S4 for gating strategy).

### Quantitative real‐time PCR and primers

Total RNA from cells was isolated using TRI Reagent (Sigma‐Aldrich) and reverse‐transcribed with RevertAid H Minus Reverse Transcriptase (Thermo Scientific) according to the manufacturer's instructions. Quantitative real‐time PCR (qRT‐PCR) was carried out using iQ SYBR Green Supermix (Bio‐Rad) and specific primers, which are listed below. The gene encoding the large ribosomal protein P0 (RPLP0) was used as a reference. Relative mRNA expression levels were calculated using the formula *x* = 2^−Δ^ *^Ct^*, where *Ct* represents the threshold cycle of a given gene and Δ*Ct* signifies the difference between the *Ct* values of the gene and the *Ct* value of the reference gene RPLP0.

Primer sequences are as follows: murine RPLP0 (detects NM_007475.5) sense, 5'‐AATCTCCAGAGGCACCATTG‐3', and antisense, 5'‐ACCCTCCAGAAAGCGAGAGT‐3'; murine IL‐31RA (detects NM_139299.2) sense, 5'‐CCCTGTGTTGTCCTGATGTTCCCA‐3', and antisense, 5'‐ACCCTTTCCAGCTTCCTCTGTCAA‐3'; murine IL‐31 (detects NM_029594.1) sense, 5'‐GCCTACCCTGGTGCTGCTTTGC‐3', and antisense, 5'‐GATGTGCTATGATGACCGAGATGTTGG‐3'.

### Histomorphology

Lung samples were fixed in 10% phosphate‐buffered formalin and marked with tissue marking dye (General Data Company Inc, Cincinnati, OH, USA) prior to paraffin embedding and processing all the samples in one tissue cassette. H&E staining was performed to evaluate basic histomorphology of the specimens. The combination of H&E and Giemsa staining highlighted the lymphocytes and eosinophils. To visualize mucopolysaccharides, Periodic acid–Schiff (PAS) staining was performed. Trichrome staining was additionally applied to visualize the collagen of the basal membrane (colored blue), which serves as a landmark for the measurement of the thickness (in μm) of the respiratory epithelium in subsegmental bronchi. The stained slides were digitized and the specimens were analyzed by an investigator blinded to the mouse groups. High‐power fields (400 × magnification corresponding to 0.066 mm^2^) were examined using a Nikon eclipse 50 microscope and a standardized image analysis system (IMS client, Imagic Bildverarbeitung AG, Glattbrugg, Switzerland). For Ki‐67 staining, immunohistochemistry was done on formalin‐fixed paraffin‐embedded (FFPE) tissue of the lung specimens according to a specialized protocol for mouse tissue [PMID: 24152994**]**. In brief, 4 μm sections were mounted on glass slides, deparaffinized with graded alcohols, and stained using the following Ki‐67‐primary antibody (Ventana, Arizona, USA), clone 30–9, rabbit monoclonal primary antibody, ready‐to‐use, 20 min). Afterwards, AffiniPure fab fragment rabbit anti‐mouse IgG (H+L) (Jackson ImmunoResearch Laboratories, Pennsylvania, USA) (dilution 1:25) was applied for 32 min. The immunohistochemical staining was performed on a Benchmark Ultra platform with the OptiView DAB IHC detection kit (both Ventana). The Ki‐67 proliferation activity was calculated by counting the number of immunohistochemically positive cells within 100 respiratory cells in each lung specimen.

### Statistical analysis

For analysis of statistical differences, one‐way ANOVA with Tukey's posthoc test was performed. Values of *p* < 0.05 were considered statistically significant: *p < 0.05, **p < 0.01, ***p < 0.001, ****p < 0.0001.

## Conflict of interest

Dr. Dillon reports that she has IL‐31‐related patents US 8647866, US 9212213, US 9738700, and US 10227415 licensed to Bristol‐Myers Squibb, and she was an employee and shareholder of Bristol‐Myers Squibb when the research was conducted. The rest of the authors declare no commercial or financial conflict of interest.

**Abbreviation**
OSM: oncostatin M

## Supporting information

Supporting Information
