# Supplementary material for: IL‐31 transgenic mice show reduced allergen‐induced lung inflammation
Source: Eur J Immunol. 2020 Jul 21;51(1):191–6. doi: 10.1002/eji.202048547 (PMC7818168; doi:10.1002/eji.202048547)
Supplement: Supplementary file 1 — Supporting Information [file EJI-51-191-s001.pdf]

Supporting Information

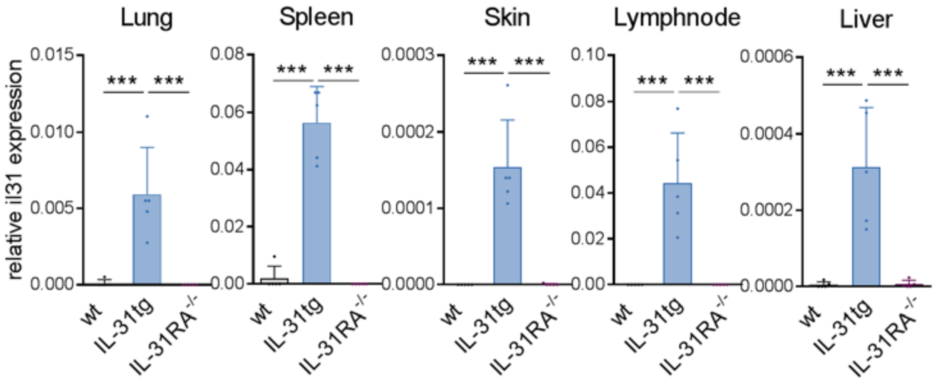

Supplementary Figure 1. Analysis of *IL31* mRNA expression in murine tissues. mRNA expression of IL-31was analyzed in lung, spleen, skin, lymph nodes and liver of naïve wild-type (wt), IL-31tg and IL-31RA$^{-/-}$ mice by qPCR. Bars represent mean+SD (n=5). One-way ANOVA with Tukey's post-hoc test was performed. ***p<0.001.

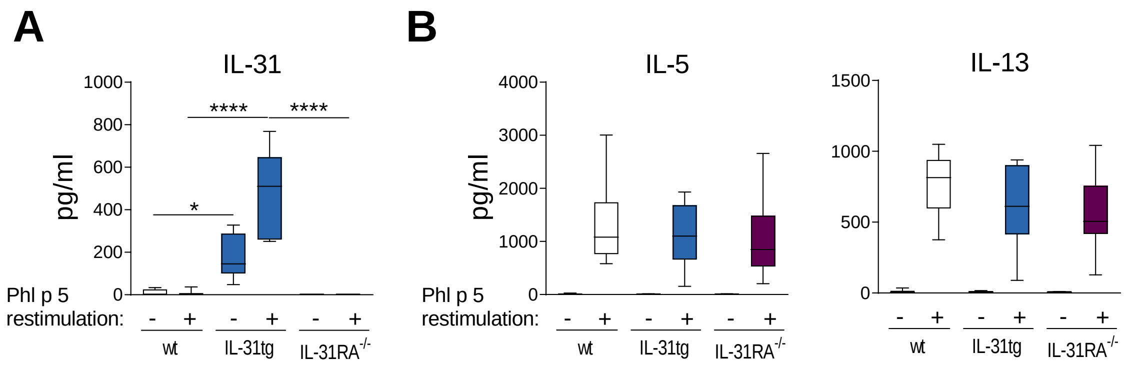

Supplementary Figure 2. Secretion of Th2-related cytokines by Phl p 5-re-stimulated splenocytes isolated from wt, IL-31tg or IL-31RA$^{-/-}$ mice is similar. Wild-type (wt), IL-31tg and IL-31RA$^{-/-}$ mice were immunized three times i.p. at 10-day intervals with Phl p 5. Ten days after the last immunization, mice were challenged intranasally on three consecutive days. The next day, splenocytes were harvested and re-stimulated with 20 µg/ml Phl p 5 for 72 h. Concentrations of **(A)** IL-31 and **(B)** IL-4, IL-5, IL-13 and IL-10 were analyzed by ELISA or multiplex assay.

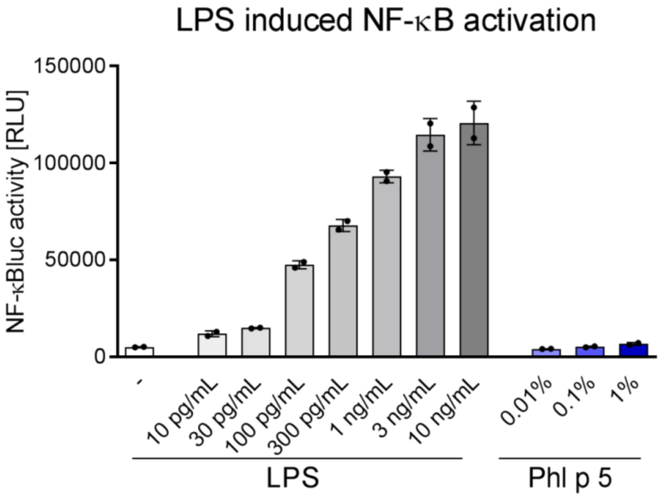

Supplementary Figure 3. Analysis of endotoxin contamination of Phl p 5. HEK293 cells were transfected with an NF-κB luciferase reporter plasmid and plasmids encoding LPS-receptor components (TLR4, CD14, MD-2). Cells were exposed to the indicated amounts of Phl p 5 or recombinant *E. coli* LPS. 20 h post-induction, luciferase activity was measured. Results show means and standard deviations of technical replicates of one experiment.

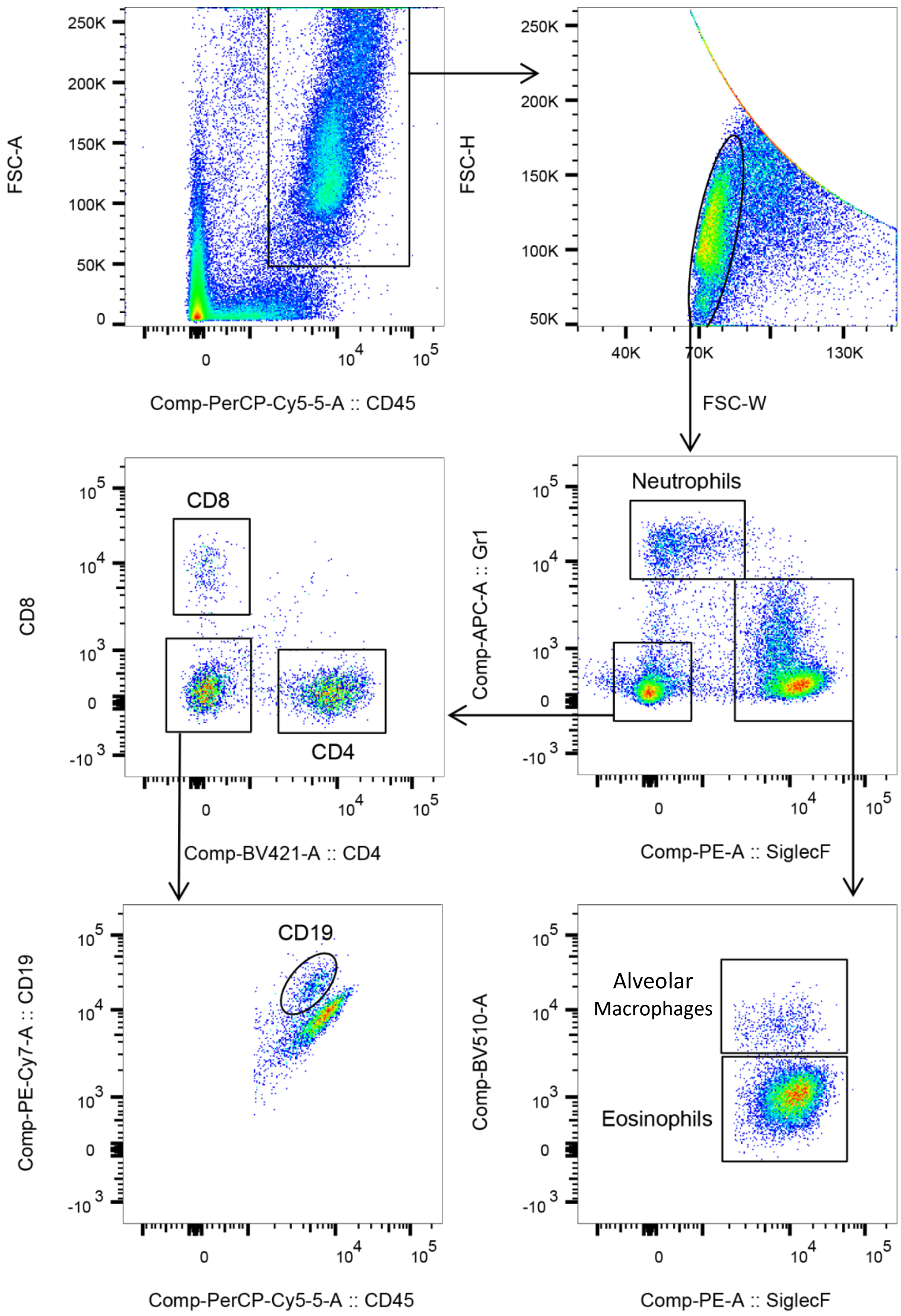

Supplementary Figure 4. Gating strategy for leukocyte analyses. Total leukocytes were gated based on their FSC and CD45 expression followed by exclusion of doublets. Neutrophils were gated as Ly6G (Gr1) high, Siglec F low cells. Siglec F high, Ly6G (Gr1) low cells were further separated into monocytes/macrophages and eosinophils based on autofluorescence in the BV510 channel. SiglecF neg Ly6G (Gr1) neg cells (containing lymphocytes) were further separated into T helper cells, cytotoxic T cells, and B cells based on their expression of lineage markers CD4, CD8a, and CD19, respectively.
